# Interleukin-6 inhibitors in non-COVID-19 ARDS: analyzing the past to step into the post-COVID-19 era

**DOI:** 10.1186/s13054-023-04394-w

**Published:** 2023-03-25

**Authors:** Lucia Gandini, Gabriele Fior, Andreas Schibler, Nchafatso G. Obonyo, Gianluigi Li Bassi, Jacky Y. Suen, John F. Fraser

**Affiliations:** 1 Critical Care Research Group, The Prince Charles Hospital, University of Queensland, Brisbane, QLD Australia; 2 Faculty of Medicine, University of Queensland, Brisbane, QLD Australia; 3 Department of Pathophysiology and Transplantation, University of Milan, Milan, Italy; 4 Intensive Care Unit, St. Andrews War Memorial Hospital, Brisbane, QLD Australia; 5 Wellcome Trust Centre for Global Health Research, Imperial College London, London, UK; 6 Initiative to Develop African Research Leaders (IDeAL)/KEMRI-Wellcome Trust Research Programme, Kilifi, Kenya

In March 2020, tocilizumab, an interleukin (IL)-6 inhibitor, was approved for the treatment of Coronavirus Disease 2019 acute respiratory distress syndrome (COVID-19 ARDS) by the Chinese National Health Commission [1]. Since then, multiple IL-6 inhibitors, including sarilumab and siltuximab, have been used off-label against COVID-19, due to the lack of effective treatments and to slow down the high pandemic mortality.

Multiple trials were conducted to assess the efficacy of IL-6 inhibitors for COVID-19 ARDS, which at times reported conflicting results. The WHO-REACT meta-analysis pooled data from 27 randomized controlled trials (RCT) on more than 10.000 COVID-19 ARDS patients showing that administration of IL-6 inhibitors, compared with usual care/placebo, was associated with lower 28-day all-cause mortality [2]. Yet, it remains unknown whether IL-6 inhibitors might have any benefit in ARDS caused by other etiologies.

To address this knowledge gap and considering the post-pandemic era, we sought to determine the effects of IL-6 inhibitors on non-COVID-19 ARDS populations. In accordance with PRISMA guidelines, we conducted a systematic review in three indexed online databases (PubMed/MEDLINE, EMBASE, CINAHL) for all pre-clinical and clinical articles examining the use of IL-6 inhibitors in non-COVID-19 ARDS, published or translated into English, up to July 18, 2022. The search terms used were a combination of headings and keywords, including three primary sets of terms pertaining to “IL-6 inhibitors/antagonists/antibodies” AND “ARDS/lung injury/respiratory failure/pneumonia/critically ill” NOT “COVID-19/SARS-CoV-2/coronavirus.” A total of 741 studies were retrieved from the initial search, resulting in 521 articles after excluding duplicates. All titles and abstracts identified were independently screened, and a total of 20 full-text published manuscripts of studies considered relevant were retrieved and independently reviewed by two authors (G.F., L.G.). Conflicting opinions were resolved by consensus with a third author (G.L.B.). Three additional studies were identified in citations during full-text screening. After excluding non-inherent articles, four studies were included in the review. Of those, only one case report was conducted in humans [3], while three RCT appraised tocilizumab effects in rat models of acute lung injury [4–6]. As shown in Table 1, tocilizumab significantly attenuated lung histopathological changes and lowered pulmonary inflammatory response and oxidative stress. In two studies [4, 5], a parallel safety trial was conducted with higher doses of tocilizumab (up to 64 mg/kg), and no adverse effect was observed. One study described a U-shaped trend of the therapeutic effect of tocilizumab [5]: doses higher than 4 mg/kg showed decreased benefit in reducing morphological lung changes. This result may be attributed to incomplete IL-6 inhibition, allowing a minimal level of signaling to maintain balance in the immune system, given the pleiotropic pro- and anti-inflammatory profile of IL-6.

**Table 1:** Summary of findings of the studies included in the systematic review

***Animal studies***									
Author	Journal, publication year	Subjects	Study design	Animals per arm	Animal model	Control and treatment groups	Objectives	Principal findings	Safety-study
Ibrahim et al.	Inflammopharmacology—Experimental Study, 2020	Rats	RCT	6	Sepsis (CLP)-induced ALI	CLP	Assess the effect of tocilizumab on sepsis-induced ALI and AKI in a sepsis rat model (CLP)	Effect of TCZ treatment	Tested doses: 4 and 8 mg/kg. No evidence of adverse effects
						CLP + TCZ 4 mg/kg (single dose)		80% reduction in 15-day mortality (*p* < 0.001)	
								Decreased serum IL-6 at 24-h (*p* < 0.001)	
								Histopathology: reduced septal thickness and inflammatory cell infiltration (*p* < 0.001); reduced lung injury scores (*p* < 0.001); reduced wet/dry ratio (*p*< 0.001)	
								BALF: lower total protein content (*p* < 0.001); lower total number of cells (*p* < 0.001); decreased TNF-a and IL-1p (*p* < 0.001)	
								Improved pulmonary oxidative and anti-oxidant profile: lipid peroxidation and total nitrite levels (*p* < 0.001); superoxide dismutase and catalase activities (*p* < 0.01); total anti-oxidant capacity (*p* < 0.001)	
								Attenuated inflammation in sepsis-induced ALI: decreased NF-kB p65 (*p* < 0.001) and p-JNK (*p* < 0.001) expression in lung tissues	
								Reduced sepsis-induced apoptosis in lung tissues: expression of Bcl-2 (*p* < 0.01), caspase-3 (*p* < 0.01)	
								Upregulation of P-glycoprotein in lung tissues (protective role against xenobiotics and endogenous compounds) (*p* < 0.001)	
Chen et al	Critical care medicine—Experimental Study, 2016	Rats	RCT	5	Severe acute pancreatic (SAP)-associated ALI	SAP + saline 0.9%	Assess the effect of tocilizumab on SAP and associated ALI in a sepsis RAT model of SAP	Effect of TCZ treatment (at 24 h)	Tested doses: 8, 16, 32, and 64 mg/kg. No evidence of adverse effects
						SAP + TCZ 2 mg/kg (single dose)		Reduced lung histological scores (*p* < 0.05)	
								Decreased lung myeloperoxidase activity, water content, and serum RAGE level in lung tissues (*p* < 0.05)	
								Improved expression of SP-A and SP-D (two members of lung surfactants related to pulmonary damage)	
Sarioglu et al	Turkish Journal of Medical Sciences—Experimental Study, 2021	Rats	RCT	5	Sepsis (LPS)-induced ALI	LPS + saline 0.9%	Assess the effects of tocilizumab ± adalimumab in intratracheal LPS-induced ALI	Effect of TCZ treatment alone and/or in combination with adalimumab	Not performed
						LPS + TCZ 10 mg/kg (single dose)		Amelioration of lung histoarchitecture (*p* < 0.001)	
						LPS + TCZ 10 mg/kg + Adalimumab 1 0 mg/kg (single dose)		Reduced lung TNF-a expression (immunoreactivity intensity) at 48-h and 96-h (*p* < 0.001)	
								Non-significant reduction in TNF-a, IL-6 and NGAL levels in BALF	

***Human studies***									
Author	Journal, publication year	Subjects	Study design	Number of patients	Protocol	Treatment	Objectives	Principal findings	Adverse effect
Petrillo et al.	Case Report Critical Care, 2020	Humans	Case report	1	–	TCZ 500 mg IV	Report a case of a patient with multiple myeloma well controlled with Carfilzomib, who was hospitalized with drug-induced ARDS and had a rapid response to TCZ	Within 24–48 h after TCZ administration	Not reported
								Vasopressor requirements lessened	
								Oxygen requirements improved	
								Laboratory evaluation revealed WBC 3.5 > 2.4 × 10^9^/L; CRP 17.7 > 3.4 mg/dL; and ferritin 2500 > 1000 ng/mL	
								Chest X-ray significant improvement in diffuse airspace opacification	

*RCT* randomized controlled trial, *CLP* cecal ligation and puncture, *ALI* acute lung injury, *TCZ* tocilizumab, *AKI* acute kidney injury, *IL-6* interleukin-6, *BALF* bronchoalveolar lavage fluid, *TNF-α* tumor necrosis factor α, *IL-1β* interleukin-1β, *NF-κB* nuclear factor-κB, *p-JNK* phosphorylated JNK, *RAGE* receptor for advanced glycation end product, *Bcl-2* B-cell lymphoma 2, *SAP* severe acute pancreatitis, *SP-D* surfactant protein D, *SP-A* surfactant protein A, *LPS* lipopolysaccharide, *CRP* C-reactive protein, *WBC* white blood cells, *NGAL* neutrophil gelatinase-associated lipocalin

In summary, this review highlights the critical lack of comprehensive evaluation of the efficacy of IL-6 inhibitors in non-COVID-19 ARDS populations. Although the identified pre-clinical studies have demonstrated effects on non-COVID-19 ARDS, similar to those observed in COVID-19 patients [2], we call attention to numerous aspects that require investigation before applying IL-6 inhibitors to non-COVID-19 ARDS patients.

First, IL-6 inhibitors were introduced for COVID-19 ARDS due to similarities between the detrimental acute inflammation observed in COVID-19 infection and the hyperinflammatory state of chimeric antigen receptor T-cell-induced cytokine release syndrome (CRS), for which IL-6 inhibitors are the approved treatment [7]. However, while the proinflammatory role of IL-6 in fueling harmful CRS is established, the role of IL-6 in ARDS pathophysiology, in which IL-6 may have also anti-inflammatory and anti-apoptotic activity [8], is not yet fully elucidated. Moreover, it remains to be determined whether IL-6 is simply a biomarker of lung injury or has a causative role in ARDS pathogenesis, and what is the harmful IL-6 threshold that justifies beginning of treatment. Considering the complexity of pathogenic pathways underlying inflammatory syndromes such as CRS and ARDS, caution is needed in translating interventions from one syndrome to the other.

Secondly, COVID-19 ARDS is an etiological subphenotype of ARDS, and results on this cohort should not be generalized to the entire ARDS population. Indeed, ARDS heterogeneity is well-recognized and several studies have shown that ARDS can be split into subphenotypes, which might respond differently to interventions [9]. A pharmacological treatment effective to lower mortality across ARDS patients remains to be identified; as such, ARDS research is now moving toward predictive enrichment. Prospective clinical trials of IL-6 inhibitors will benefit by following this trend and selectively targeting specific ARDS subphenotypes, rather than trialing an unselected population.

Third, in most previous COVID-19 studies, it is difficult to determine the ARDS phase (exudative/inflammatory, proliferative, fibrotic) in which IL-6 inhibitors have been administered, leaving best timing for treatment still to be determined. Indeed, during pandemic, hospitals were overwhelmed, and patients have been treated at various ARDS stages.

Fourth, each of the several IL-6 inhibitors available has a different target in the IL-6 cascade. Precisely, IL-6 has three distinct signaling pathways (classic, trans-signaling, trans-presentation) and drugs interfere with them at different levels [10]. For example, direct IL-6 antagonists (e.g., siltuximab) inhibit the classic pathway, while inhibitors of IL-6 membrane receptors (e.g., tocilizumab, sarilumab) block all the three pathways. No study has yet compared the different efficacies of these drugs in ARDS. What is the most efficacious IL-6 inhibitor? What is the correct dose? Single or multiple doses? Answers to these questions are warranted.

Finally, IL-6 inhibitors have historically been used in chronic inflammatory diseases. Shifting the indication of this class of immunomodulators to an acute disease, such as ARDS, needs to cautiously consider the higher risk of adverse effects in critically ill patients. So far, data on secondary infections are limited and definitions of adverse events were not consistent across COVID-19 trials.

To the best of our knowledge, robust data on effects of IL-6 inhibitors in non-COVID-19 ARDS are not available, and no ongoing clinical study can be found on clinicaltrials.gov/WHO trials registry/Cochrane trials registry (search updated to July 18, 2022). While appreciating the treatment opportunity these immunomodulators could exercise in the post-pandemic era, high-quality mechanistic studies and subphenotype-targeted prospective trials are now warranted before generalizing to the entire ARDS population promising results obtained during the pandemic.

## Data Availability

The datasets about the strategies of search used during the current study are not publicly available because we could not include them in the manuscript of this article type. However, they are available from the corresponding author on request.
